# Acknowledgement of manuscript reviewers 2015

**DOI:** 10.1186/s12942-016-0040-1

**Published:** 2016-03-09

**Authors:** Maged N Kamel Boulos

**Affiliations:** University of the Highlands and Islands, Inverness, UK

**Geir Aamodt**

Norway

**Philip Abdelmalik**

Canada

**C N Agoti**

Kenya

**J Aldstadt**

USA

**N Alexander**

Colombia

**L Arbuckle**

Canada

**Martijn Arns**

Netherlands

**Vishal Arora**

UK

**A Bandyopadhyay**

USA

**D Berrigan**

USA

**Janne Boone-Heinonen**

USA

**Manfred Buchroithner**

Germany

**Anibal Carbajo**

Argentina

**Marilia Carvalho**

Brazil

**Steve Carver**

UK

**Neil Coffee**

Australia

**M Cohen**

USA

**Natalie Colabianchi**

USA

**Fabrice Courtin**

France

**D F Cuadros**

Qatar

**Andrew Curtis**

USA

**Paul Delamater**

USA

**Kenneth Denike**

Canada

**N Deroche**

South Africa

**James Dunn**

Canada

**Lutz Ehlkes**

Germany

**Khaled El Emam**

Canada

**Jean-Philippe Empana**

France

**Chellafe Ensoy**

Belgium

**Roger Evans**

UK

**Abby Flynt**

USA

**J Gibbons**

USA

**Jianya Gong**

China

**William B Grant**

USA

**G Grekousis**

Greece

**Aline Guttmann**

France

**Michael Hagenlocher**

Germany

**K Herbst**

South Africa

**C Herrmann**

Switzerland

**Gary Higgs**

UK

**James Holt**

USA

**Douglas Houston**

USA

**Rebekah Huber**

USA

**Andrew Hudson-Smith**

UK

**Alvaro Iruin**

Spain

**Enrico Ivaldi**

Italy

**M Jankowska**

USA

**Caroline Jeffery**

UK

**Jeremie Jegu**

UK

**S Y Jeong**

Republic of Korea

**Y Jin**

USA

**Andy Jones**

UK

**Rena Jones**

USA

**I Jung**

Republic of Korea

**Lucie Kalousova**

USA

**M N Kamel Boulos**

USA

**Steve Kammerer**

USA

**N Kapusta**

Austria

**H Karimi**

USA

**P Kasemsuppakorn**

Thailand

**A Klassen**

USA

**Christian Klaus**

USA

**Tom Koch**

Canada

**M Kondo**

USA

**O Kounadi**

Austria

**Petr Kubicek**

Czech Republic

**Martin Kulldorff**

USA

**Mei-Po Kwan**

USA

**J Larmarange**

France

**Ryan Lash**

USA

**Veerle Lejon**

France

**Liliana Leone**

Italy

**Daniel Lewis**

UK

**Yan Lin**

USA

**R Lipton**

USA

**Jonathan London**

USA

**Valerie R Louis**

Germany

**Hui Luan**

Canada

**S Macrury**

UK

**R Maheswaran**

UK

**Alessandro Mannelli**

Italy

**Liang Mao**

USA

**I Maramba**

Uk

**M Marlow**

USA

**Tony Mathys**

UK

**S Mazumdar**

Australia

**Paul Mccrorie**

UK

**Noreen Mcdonald**

USA

**Moran Mika**

Israel

**Michelle Morris**

UK

**Sarah-Anne Munoz**

UK

**Bronwyn Myers**

Australia

**Keiko Nakamura**

Japan

**Robin Nesbitt**

Germany

**Dennis Nicholas**

USA

**Oliver O’Brien**

UK

**Lisel O’Dwyer**

Australia

**G Onicescu**

USA

**S Orton**

UK

**A Pascoe**

UK

**L Peipins**

USA

**R Peled**

Israel

**Gavin Pereira**

Australia

**A Perez-Navarro**

Spain

**Revati Phalkey**

Germany

**Hugo Pilkington**

France

**B Piot**

USA

**Andrea Presotto**

USA

**Matthew Quick**

Canada

**G Ramjee**

South Africa

**Bernd Resch**

Germany

**Jennifer Roberts**

USA

**J Rodas**

Colombia

**Francisco Miranda Rodrigues**

Portugal

**Mark Rosenberg**

Canada

**Corrine Ruktanonchai**

UK

**Andrew Rundle**

USA

**Gerard Rushton**

USA

**Paul Salze**

France

**G Sanguinetti**

UK

**Nadine Schuurman**

Canada

**Vincent Seaman**

USA

**Safraj Shahul Hameed**

India

**Wei Shi**

Canada

**T Stübig**

Germany

**Wayne Sullender**

USA

**H Tao**

China

**S D Towne**

USA

**Gisela Van Kessel**

Australia

**Jean-Francois Viel**

France

**Ari Voutilainen**

Finland

**John Vulule**

Kenya

**Edward Walker**

USA

**Neng Wan**

USA

**Fahui Wang**

USA

**Tai-Chi Wang**

Taiwan

**Hannah Weir**

USA

**Laura White**

USA

**Michael Widener**

Canada

**F Williams**

USA

**Michael Wimberly**

USA

**M Winters**

Canada

**C Yu**

China

**C-S Yue**

Taiwan

**Xingyou Zhang**

USA

**Li Zhu**

USA

**Marsil Zook**

Australia

